# Culturing Keratinocytes on Biomimetic Substrates Facilitates Improved Epidermal Assembly In Vitro

**DOI:** 10.3390/cells10051177

**Published:** 2021-05-12

**Authors:** Eve Hunter-Featherstone, Natalie Young, Kathryn Chamberlain, Pablo Cubillas, Ben Hulette, Xingtao Wei, Jay P. Tiesman, Charles C. Bascom, Adam M. Benham, Martin W. Goldberg, Gabriele Saretzki, Iakowos Karakesisoglou

**Affiliations:** 1 Department of Biosciences, Durham University, Durham DH1 3LE, UK; eve.f.hunter-featherstone@durham.ac.uk (E.H.-F.); natalie.young@durham.ac.uk (N.Y.); kathryn.chamberlain@durham.ac.uk (K.C.); adam.benham@durham.ac.uk (A.M.B.); m.w.goldberg@durham.ac.uk (M.W.G.); 2 Department of Earth Sciences, Durham University, Durham DH1 3LE, UK; pablo_cubillas@hotmail.com; 3 The Procter & Gamble Company, Cincinnati, OH 45202, USA; hulette.bc@pg.com (B.H.); wei.x.3@pg.com (X.W.); tiesman.jp@pg.com (J.P.T.); bascom.cc@pg.com (C.C.B.); 4 Biosciences Institute, Newcastle University, Newcastle-upon-Tyne NE1 7RU, UK; gabriele.saretzki@newcastle.ac.uk

**Keywords:** mechanotransduction, LINC complex, nesprin, Sun-domain protein, nuclear lamina, lamin, keratinocytes, skin biomechanics, biomimetic dishes, skin equivalents

## Abstract

Mechanotransduction is defined as the ability of cells to sense mechanical stimuli from their surroundings and translate them into biochemical signals. Epidermal keratinocytes respond to mechanical cues by altering their proliferation, migration, and differentiation. In vitro cell culture, however, utilises tissue culture plastic, which is significantly stiffer than the in vivo environment. Current epidermal models fail to consider the effects of culturing keratinocytes on plastic prior to setting up three-dimensional cultures, so the impact of this non-physiological exposure on epidermal assembly is largely overlooked. In this study, primary keratinocytes cultured on plastic were compared with those grown on 4, 8, and 50 kPa stiff biomimetic hydrogels that have similar mechanical properties to skin. Our data show that keratinocytes cultured on biomimetic hydrogels exhibited major changes in cellular architecture, cell density, nuclear biomechanics, and mechanoprotein expression, such as specific Linker of Nucleoskeleton and Cytoskeleton (LINC) complex constituents. Mechanical conditioning of keratinocytes on 50 kPa biomimetic hydrogels improved the thickness and organisation of 3D epidermal models. In summary, the current study demonstrates that the effects of extracellular mechanics on keratinocyte cell biology are significant and therefore should be harnessed in skin research to ensure the successful production of physiologically relevant skin models.

## 1. Introduction

Skin is the largest human organ acting as the primary interface with the peripheral environment; providing protection from ultraviolet (UV) radiation, maintaining water homeostasis and acting as the first point of immune surveillance [1]. As the upper layer of the skin, the epidermal compartment, composed predominantly of keratinocytes, is exposed to many external stressors including mechanical manipulation. A hallmark of epidermal biology is a high cell turnover, which allows for constant re-generation and efficient wound healing. Using various mechanosensory proteins, keratinocytes are able to respond to sheer forces, stretch and compression through changes to their proliferation, migration, and differentiation rates [2,3,4].

Mechanosensation involves cell adhesion molecules such as integrins and cadherins. Integrins mechanically attach cells to the extracellular matrix (ECM), whilst E-cadherin is a key cell-cell adhesion molecule in the epidermis. Upon coming into contact with ECM components or neighbouring cells, integrins and cadherin molecules use localised contractions to assess the rigidity of their respective binding partners [5,6,7]. Integrins are anchored to cytoplasmic actin filaments by vinculin and talin, while E-cadherin associates to the actin cytoskeleton via α- and β-catenin. Using myosin IIB filaments, cells are able to contract these adhesion molecules and assess the rigidity of their binding partners based on the level of force required to induce displacement of either the ECM or neighbouring cells [6,7]. Whilst the mechanism is not fully understood, E-cadherin contraction is deemed essential to the formation of epithelial layers due to its role in controlling monolayer organisation [7]. E-cadherin-dependent epithelial cell adhesion has been shown to alter in response to changes in Young’s modulus of polyacrylamide gel substrates in cell culture, leading to differences in cytoskeletal organisation and cell morphology [8].

Engagement of integrins at the epidermal basement membrane relays high tension to the nucleus via the Linker of Nucleoskeleton and Cytoskeleton (LINC) complex; reportedly resulting in repression of differentiation and maintenance of keratinocyte progenitor cells [9]. The LINC complex is comprised of the evolutionarily conserved nesprin and the Sun-domain proteins that span the nuclear envelope. Nesprins associate with the cytoskeleton at the outer nuclear membrane, while their conserved C-terminal KASH-domain binds the C-terminal half of the inner nuclear membrane Sun-domain proteins in the nuclear envelope lumen. The N-termini of Sun-domain proteins protrude into the nucleoplasm and associate with nucleoskeletal structures. Thus, the LINC complex connects the cytoskeleton to the nuclear lamina and chromatin, enabling the communication of external mechanical cues directly to the nuclear interior [10,11,12,13]. This cell-spanning protein network controls nuclear positioning, cell adhesion, actin dynamics and directed cell migration in response to local mechanosensory information [14,15,16]. In skin, the LINC complex plays a role in epidermal organisation [17] and cell fate regulation, with integrin adhesion to the basement membrane being the primary determinant [18]. Culture on micro-patterned substrates has revealed that the degree of integrin-mediated adhesion is directly correlated to the level of differentiation; decreased adhesive area being associated with terminally differentiated cells, whilst high expression of integrin α6 and β1 subunits is observed in epidermal stem cells [19,20]. This transition from a proliferative to a differentiated phenotype has been attributed to the degree of tension placed on the nuclear lamina, particularly lamin A. In keratinocytes with high levels of integrin-mediated adhesion, the tension on lamin A is high, but migration away from the basement membrane results in the loss of these focal adhesions and relaxation of lamin A tension [9]. The role of lamins in epidermal differentiation is further consolidated by a study on a skin-specific triple lamin knockout mouse model (*Lmna−/− Lmnb1Δ/Δ Lmnb2Δ/Δ*), which exhibit precocious epidermal differentiation, evidenced by thickening of the epidermis and hyperkeratosis [21].

A key mechanosensitive pathway is the Hippo signalling pathway, which controls the balance between keratinocyte proliferation and differentiation [22]. Hippo pathway activation has been shown to be controlled by mechanical stimuli. Where the external environment is “soft”, the mechanical stimulus is low; activating the pathway so that the transcription factors Yes-associated protein (YAP) and transcriptional coactivator with PDZ-binding motif (TAZ) are sequestered to the cytoplasm, resulting in growth arrest and differentiation. In a stiff environment, YAP/TAZ are activated and localise to the nucleus, resulting in the upregulation of genes related to cell proliferation [23,24]. Epithelia primed on stiff substrates retain their cell adhesion and migratory properties when exposed to softer matrixes by exhibiting YAP-dependent mechanical memory [25]. Similarly, human mesenchymal stem cells retain a memory of past mechanical environments, which affects future cell fate decisions [26]. In the epidermis, proliferative cells are found in the basal layer where they sit on a stiff basement membrane [27]. As the keratinocytes move away from the basement membrane into the suprabasal layer and beyond, the mechanical stimuli are altered, prompting a switch from a proliferative phenotype and keratinocyte stemness, and driving cells towards differentiation [27].

Despite the evident importance of external mechanics on cell behaviour [28], conventional in vitro cell culture relies heavily on extremely stiff substrates, such as plastic and glass, which do not reflect the mechanics of the physiological microenvironment. Young’s modulus, or elastic modulus, describes the relationship between stress (force divided by area) and strain (change in shape as a result of stress) of a material, with stiffer materials being associated with a high elastic modulus [29]. Whilst the literature does not provide an exact value for the stiffness of the epidermis, human skin exhibits a Young’s Modulus range of low to mid kilopascals (kPa) (0.1–10 kPa) [30]. However, tissue culture plastic (TCP) possesses a Young’s Modulus in the Gigapascal (GPa) range [31]. Given that cells utilise adhesion molecule contractions to determine the stiffness of their surroundings through material displacement [5,6,7], it is striking that TCP and glass are still used in cell culture. Recent advances in three-dimensional (3D) tissue model development enable cells to construct their own 3D microenvironment through ECM deposition [32]. Despite this, in the majority of cases, cells are first being primed on a two-dimensional (2D) TCP surface. Moreover, in vitro skin models have been shown to have a gene expression profile similar to hyperproliferative skin, with marked upregulation of keratin 16; usually, a hallmark of keratinocyte activation and observed at wound sites and in psoriatic epidermis [33,34]. Consequently, current in vitro skin models are not fully representative of native skin.

The purpose of this study was to directly compare the physiology and capacity for epidermal assembly of primary human keratinocytes cultured on TCP, and biomimetic hydrogels (BMH) designed to replicate the mechanical properties of the epidermis. Through a combination of microscopy, protein, and functional analysis, keratinocyte morphology, proliferative capacity, cytoskeletal arrangement, and expression of key mechanoproteins were monitored. Keratinocytes cultured on BMH showed increased colony density, changes in proliferation, and expression of key mechanoproteins such as nesprins-1/-2, Sun1 and lamins. Moreover, when primed on BMH, keratinocytes demonstrated superior epidermal assembly in the form of thicker and more organised 3D epidermal equivalents.

## 2. Materials and Methods

### 2.1. Cell Culture

Human epidermal keratinocytes, neonatal (HEK; LifeLine Cell Technology, Oceanside, CA, USA) were cultured as a monolayer on 10 cm diameter dishes of either standard TCP (Greiner BioOne, Stonehouse, UK) or coated with a 4, 8 or 50 kPa collagen-coated biomimetic hydrogel (Petrisoft^®^, Cell Guidance Systems, Cambridge, UK). Cells were maintained in EpiLife medium containing 60 μM calcium supplemented with Human Keratinocyte Growth Supplement (HKGS), 0.25 µg/mL amphotericin B and 10 µg/mL gentamicin. All cell culture reagents were Gibco™ (ThermoFisher Scientific, Loughborough, UK). 2D cultures were kept at 37 °C in a 5% CO_2_ humidified incubator following the supplier’s instructions. Cells in 2D culture were imaged using an EVOS XL Core Cell Imaging System.

Three-dimensional (3D) epidermal cultures were carried out using BRAND^®^ insert strips for 24 × 6-well BRANDplates^®^ (polycarbonate, 0.4 µm pore size) with the 6-well BRANDplates^®^ (Sigma-Aldrich, Gillingham, UK). Inserts were coated with human collagen I diluted 1:100 (Coating Matrix Kit protein; ThermoFisher Scientific, Loughborough, UK) 30 min prior to use. HEK were dissociated from their respective dishes using TrypLE express enzyme, and resuspended in EpiLife medium containing 60 μM CaCl_2_ supplemented with HKGS, 0.25 µg/mL amphotericin B, 10 µg/mL gentamicin, and 10 ng/mL of keratinocyte growth factor (KGF). All cell culture reagents were obtained from Gibco™ (ThermoFisher Scientific, Loughborough, UK). Cells were seeded at a density of 2.5 × 10^5^ cells per insert and incubated at 37 °C in a 5% CO_2_ humidified incubator for 4 days, before being raised to the air-liquid interface and further supplemented with 25 µg/mL ascorbic acid (Sigma-Aldrich, Gillingham, UK) and 1.5 mM CaCl_2_ and maintained for an additional 12 days.

### 2.2. Histological Analysis

Epidermal equivalents were fixed in 10% formalin (Sigma-Aldrich, Gillingham, UK) in PBS overnight at 4 °C then gradually dehydrated in 30–100% ethanol and Histoclear (ThermoFisher Scientific, Loughborough, UK) before being embedded in paraffin wax using dispomoulds (CellPath, Newton, UK). All wax blocks were sectioned at 7 μm using a Leica RM2125RT microtome and mounted onto Superfrost plus microscope slides (4951PLUS4, ThermoFisher Scientific, Loughborough, UK).

For haematoxylin and eosin (H and E) staining, slides were deparaffinised in Histoclear then gradually rehydrated in ethanol (100–70%) and deionised water. The slides were incubated in Mayer’s haematoxylin (Sigma-Aldrich, Gillingham, UK) for 5 min, then submerged in deionised water followed by alkaline ethanol for 30 s. Samples were once again dehydrated through submersion in ethanol before being incubated in eosin (Sigma-Aldrich, Gillingham, UK) for 20 s and further dehydrated in ethanol and Histoclear. Slides were mounted using DPX (Sigma-Aldrich, Gillingham, UK) and imaged using a Leica ICC50 high-definition camera mounted onto a Brightfield Leica microscope.

### 2.3. Immunostaining

For 2D immunostaining of cells, HEK were cultured on 12 mm diameter glass coverslips (Scientific Laboratory Supplies (SLS), Wilford, UK) or biomimetic (Softslip^®^, Cell Guidance Systems, Cambridge, UK) coverslips in a 24-well plate for 4 days in 1 mL EpiLife medium containing 60 μM CaCl_2_ supplemented with HKGS, 0.25 µg/mL amphotericin B and 10 µg/mL gentamicin, and incubated in a humidified atmosphere with 5% CO_2_ at 37 °C. Cells were fixed in 3.7% formalin in PBS for 15 min at room temperature then washed three times in Dulbecco’s phosphate-buffered saline (DPBS). Permeabilisation, if required, was performed by incubating coverslips in 0.5% Triton-X-100 in PBS for 10 min at room temperature. Blocking solution was composed of 1% bovine serum albumin (BSA, Sigma-Aldrich, Gillingham, UK) and 0.1% fish gelatin (Sigma-Aldrich, Gillingham, UK) in PBS. Coverslips were incubated with primary antibodies (Table S1) in a humid atmosphere for 1 h at room temperature, followed by three washes in TBS (50 mM Tris-HCl, pH 7.6; 150 mM NaCl) + 0.1% Tween 20 (TBST). Coverslips were incubated for 1 h at room temperature with the secondary antibodies (Table S2) and 2 µg/mL DAPI (Sigma-Aldrich, Gillingham, UK), then washed again three times in TBST. Coverslips were mounted onto slides using of VECTASHIELD^®^ anti-fade mounting medium (H1000; Vector Laboratories, Peterborough, UK) then imaged using a Zeiss LSM 880 with Airyscan (Zeiss, Cambridge, UK).

For 3D immunostaining of epidermal models, slides were deparaffinised in Histoclear and gradually rehydrated in ethanol and deionized water. Antigen retrieval was achieved by incubating slides in citrate buffer (0.1 M, pH 6.0) at 98 °C for 15 min, and once cooled samples were blocked in a humidified box for 1 h at room temperature in 10% goat serum and 0.1% BSA (Sigma-Aldrich, Gillingham, UK) in PBS. Primary antibodies (Table S1) were diluted in 1:60 goat serum (Abcam, Cambridge, UK) and 0.1% BSA in PBS and incubated with samples overnight at 4 °C. Slides were washed three times in TBST then incubated for 60 min at room temperature with the secondary antibodies (Table S2) and 2 µg/mL DAPI (Sigma-Aldrich, Gillingham, UK) diluted in 1:60 goat serum and 0.1% BSA in PBS. Slides were mounted using VECTASHIELD^®^ anti-fade mounting medium (Vector Laboratories, Peterborough, UK) and a glass coverslip, and imaged using a Zeiss LSM 880 with Airyscan.

### 2.4. EdU Proliferation Assay

HEK were cultured on either glass (SLS, Wilford, UK) or biomimetic (Cell Guidance Systems, Cambridge, UK) coverslips for a minimum of 4 days ensuring that confluency did not surpass 60%. The EdU assay was performed using the Click-iT^®^ EdU Alexa Fluor^®^ 488 kit (Invitrogen, ThermoFisher Scientific, Loughborough, UK), using the recommended protocol. Medium was removed and replaced with fresh medium containing 10 μM EdU, and cells were incubated for 30 min in a humidified atmosphere with 5% CO_2_ at 37 °C. Coverslips were fixed in 10% formalin then washed twice in 3% BSA in PBS before being permeabilised following the immunofluorescence protocol above. 0.5 mL of Click-iT^®^ reaction cocktail was added to each coverslip and incubated at room temperature for 30 min. Nuclei were labelled using 2 µg/mL DAPI (Sigma-Aldrich, Gillingham, UK) and coverslips mounted with VECTASHIELD^®^ anti-fade mounting medium (Vector Laboratories, Peterborough, UK) and imaged using a Zeiss LSM 880 with Airyscan (Zeiss, Cambridge, UK).

### 2.5. Quantification of Nuclear and Cytoplasmic YAP1 Staining

Primary HEK cells were grown on TCP and 4 kPa coverslips for 4 days before being processed for YAP1 immunofluorescence. In addition, cells were counterstained with fluorescently labelled phalloidin and DAPI. All samples were documented under identical imaging conditions using confocal microscopy (Zeiss LSM 880 with Airyscan). In order to assess the intensity of staining in the cytoplasmic and nuclear compartments of the cells, maximum intensity images were analysed using the image processing software Image J/Fiji. The nucleus and cytoplasmic regions of the cells of interest were selected using the freehand selection tool and “area integrated density” was measured. The average Integrated Density was calculated and used to plot the presented graphs. Integrated density is the product of area and mean grey value enabling the analysis of both bright and dim pixels within an image. The aforementioned procedure is a more reliable measurement of staining intensity than Mean Grey Value alone. In total 165 cells per each condition were analysed.

### 2.6. Osmotic Shock Assay

HEK were cultured on either glass (SLS, Wilford, UK) or 4 kPa biomimetic (Cell Guidance Systems, Cambridge, UK) coverslips for a minimum of 4 days. Medium was removed and replaced with fresh medium containing 640 mM sucrose and cells were incubated for 30 min in a humidified atmosphere with 5% CO_2_ at 37 °C. Coverslips were fixed in 10% formalin then washed in PBS before being permeabilised following the immunofluorescence protocol above. Cells were stained for the nuclear envelope marker lamin B1 (Table S1) and nuclei were labelled using 2 µg/mL DAPI and coverslips were mounted with VECTASHIELD^®^ anti-fade mounting medium (Vector Laboratories, Peterborough, UK) and imaged using a Zeiss LSM 880 with Airyscan. This enabled the percentage of cells exhibiting nuclear abnormalities, defined as clear folds/creases in the envelope, to be calculated.

### 2.7. Western Blotting

Two-dimensional (2D) cultured cell lysates were obtained by incubating cell cultures on a rocker on ice for 30 min in RIPA lysis buffer (50 mM Tris, 150 mM NaCl, 0.1% SDS, 1% Nonidet P-40, 0.5% Sodium-deoxycholate, 1% Protease Inhibitors (Pierce™ mini-tablets, ThermoFisher Scientific, Loughborough, UK)), before being removed using a cell scraper (VWR, Leicestershire, UK). The resulting lysates were then sheared by passing them through a 23G needle (BD Microlance™; ThermoFisher Scientific, Loughborough, UK) 20 times and centrifuged at 4 °C (10 min, 13,000× *g*). The supernatants were mixed 1:5 with sample loading buffer (Laemmli buffer containing 5% 2-mercaptoethanol) and heated at 98 °C for 4 min to denature proteins.

Samples were separated on 10% Tris-Glycine SDS-PAGE gels (for proteins above 250 kDa, Novex™ 4–12% Tris-Glycine gradient gels were used (ThermoFisher Scientific, Loughborough, UK)). Proteins were transferred onto methanol primed Immobilon^®^-P PVDF transfer membrane (Merck Millipore, Watford, UK) and then membranes were blocked for 45 min in 5% milk in TBST. Primary antibodies (Table S1) were diluted in 5% milk and incubated overnight at 4 °C, membranes were then washed three times in TBST and incubated with secondary antibodies (Table S2) for 1 h at room temperature. Membranes were either developed using the Amersham ECL Prime Western Blotting Detection Reagent (GE Healthcare Life Sciences, Little Chalfont, UK), exposed to film and developed using an X-OMAT X-ray developer; or Clarity Western ECL Substrate (BioRad, Watford, UK) and detected using an iBright imaging system (ThermoFisher Scientific, Loughborough, UK). The relative levels of proteins were determined by densitometry, with data normalised to the respective loading controls; GAPDH and ß tubulin, and analysed using Fiji [35].

### 2.8. Atomic Force Microscopy (AFM)

HEK were cultured on TCP (Greiner BioOne, Stonehouse, UK) or 4 kPa Petrisoft^®^ biomimetic 10 cm dishes for 4 days following the standard protocol previously described. Cells were then trypsinised and transferred to the lids of TCP 6 cm dishes (Greiner BioOne) and allowed to adhere overnight. Dish lids were used at this stage due to their lower sides, which were required to ensure samples fitted into the AFM. The ability of cells to adhere to dish lids was tested prior to running the experiments. In order to ensure adequate provision of medium, the lids were placed within a standard 10 cm dish overnight before being removed in order to conduct the experiment. Cells were analysed using a NanoWizard^®^ 3 Bioscience AFM (JPK) using a Silicon Nitride pyramidal probe cantilever with a spring constant of 0.005–0.022 Nm^−1^ (AppNano, Mountain View, CA, USA). Young’s modulus values were assessed across 10 different cytoplasmic and nuclear regions per cell using JPKSPM Data Analysis software and the supplied Hertz-Fit Application Note for biological samples. In total 6 random cells for each condition (i.e., TCP and 4 kPa) were analysed.

### 2.9. Statistical Analysis

Statistical analysis was calculated using GraphPad Prism v9 (GraphPad, San Diego, CA, USA); with statistical significance taken at *p* ≤ 0.05, and data presented as mean ± SEM. A one-way ANOVA (Analysis of variance) was used to determine significance with a Tukey’s post hoc test for multiple comparisons, and a Dunnett’s post hoc test for single comparisons to control (TCP). For comparisons between two groups, e.g., F-actin, plectin, YAP1 (Figure 2), and lamin B1 immunofluorescence and basal cell analysis (nuclear height and density), an unpaired t-test was used.

## 3. Results

In this study, we report the effects of culturing primary HEK on BMH that mimic the in vivo epidermal environment, rather than standard TCP, on the cell biology of HEK cells and on the formation of epidermal skin equivalent models. Cells were primed on TCP and 50, 8 and 4 kPa substrates before being investigated for changes in cell architecture, mechanoprotein levels and ability to assemble 3D epidermal equivalents. The stiffness values for the BMH conditions were selected based on Young’s Modulus values given for skin and other relevant basement membranes in the literature [30,36,37,38,39,40].

### 3.1. Characterisation of HEK Behaviour on TCP and BMH Surfaces

**Figure 1:**
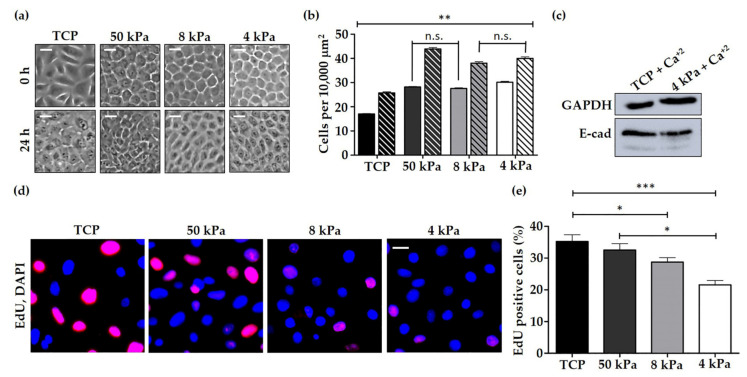
HEK cultured on BMH exhibit increased cell density and decreased proliferation. (**a**) Representative bright-field images of HEK cultured on TCP and BMH, 0 and 24 h after treatment with 1.5 mM CaCl_2_. Scale bars: 20 µm. (**b**) Quantification of cell density in a defined cell colony area of 10,000 µm^2^ for each culture dish 0 and 24 h after CaCl_2_ treatment (Solid columns = 0 h 1.5 mM CaCl_2_, Striped columns = 24 h 1.5 mM CaCl_2_). Data represent mean ±SEM, *n* = 3, statistical significance was assessed using one-way ANOVA with Tukey’s post hoc test, n.s. = non-significant (i.e., 50 kPa versus 8 kPa [low Ca^+2^]; 8 kPa versus 4 kPa [high Ca^+2^]), ** *p* ≤ 0.001. (**c**) Western blot of E-cadherin expression in HEK cultured on TCP and 4 kPa BMH following 1.5 mM CaCl_2_ treatment. GAPDH levels indicate equal loading of proteins. (**d**) Representative confocal microscopy images of EdU proliferation assay on HEK cultured on TCP and BMH. DAPI stain denotes nuclei. Scale bars: 20 µm. (**e**) Quantification of the percentage of EdU positive cells grown on TCP and BMH cell culture dishes. Data represent mean ±SEM, *n* = 3, statistical significance was assessed using one-way ANOVA with Tukey’s post hoc test, * *p* ≤ 0.05, *** *p* ≤ 0.0001.

HEK were cultured on either TCP or BMH cell culture dishes then supplemented with 1.5 mM CaCl_2_ to replicate the higher end of the epidermal calcium gradient, and observed 0 and 24 h later. At 0 h, HEK on BMH were noted to form colonies exhibiting the characteristic cobblestone morphology expected from primary keratinocytes in vitro (Figure 1a). This organisation and regularity of cell shape was strikingly less apparent in HEK cultured on standard TCP. At 24 h after CaCl_2_ addition, HEK exhibited denser packing on BMH, particularly 50 kPa and 4 kPa, HEK cells appeared considerably smaller and more crowded (Figure 1a). To assess cell density changes across the conditions, the number of cells within a colony per 10,000 µm^2^ was quantified. HEK cultured on BMH were found to have a higher cell density than those on TCP at both 0 and 24 h after CaCl_2_ supplementation (Figure 1b). Western blot analysis was used to examine the levels of the cell-cell adhesion protein E-cadherin in HEK cultured on TCP and 4 kPa BMH in high calcium conditions (1.5 mM CaCl_2_). High calcium conditions were used here as E-cadherin becomes the dominant cadherin in adherens junctions as keratinocytes undergo differentiation [41], however, there was no obvious change in E-cadherin levels (Figure 1c and Figure S1).

**Figure 2:**
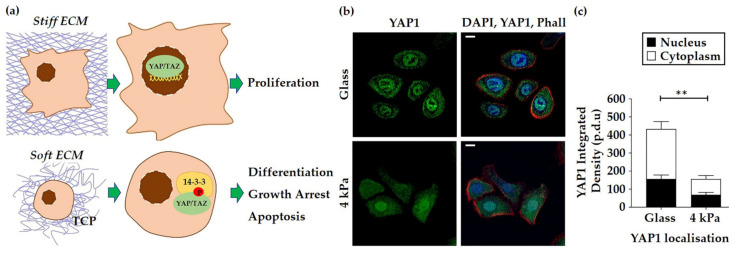
HEK cultured on BMH exhibit altered YAP1 localisation. (**a**) Activation or deactivation of the Hippo signalling pathway on a stiff or soft extracellular matrix (ECM) alters the localisation of YAP/TAZ (nuclear versus cytoplasmic) and determines keratinocyte proliferation or differentiation. (**b**) Representative images of YAP1 localisation in HEK cultured on glass and 4 kPa coverslips. DAPI was used to stain the nucleus and Alexa Fluor-568 conjugated phalloidin to detect F-actin. Scale bars: 10 µm. (**c**) Quantification of integrated density of YAP1 in the nuclear and cytoplasmic compartments of HEK on glass and 4 kPa BMH coverslips. Data represent mean ± SEM, *n* = 3, statistical significance was assessed using an unpaired *t*-test, ** *p* ≤ 0.001. Both the total YAP1 integrated density as well as the individual YAP1 integrated intensities measured within the nuclear and cytoplasmic compartments were significantly reduced in cells grown on 4 kPa coverslips.

Another clear distinction between HEK grown on TCP and BMH was the difference in proliferation rate. An EdU assay revealed that HEK cultured on TCP proliferated significantly more than those on 4 and 8 kPa BMH substrates (Figure 1d,e). The Hippo signalling pathway is activated when cells are exposed to a soft ECM, causing YAP/TAZ to be sequestered in the cytoplasm or degraded, therefore resulting in decreased proliferation (Figure 2a). This aligns with the changes in proliferative capacity observed in this study. Moreover, immunofluorescence (IF) analysis of YAP1 in HEK revealed that the integrated density of the staining was greater in the nuclei and cytoplasm of cells cultured on glass coverslips rather than 4 kPa BMH (Figure 2b,c). In addition, overall integrated density of YAP1 was greater for HEK on TCP, suggesting that there was more YAP1 protein present in cells cultured on TCP (Figure 2c).

### 3.2. HEK Alter Their Cytoskeletal Organisation and Nuclear Mechanics in Response to Cell Culture on Softer Substrates

**Figure 3:**
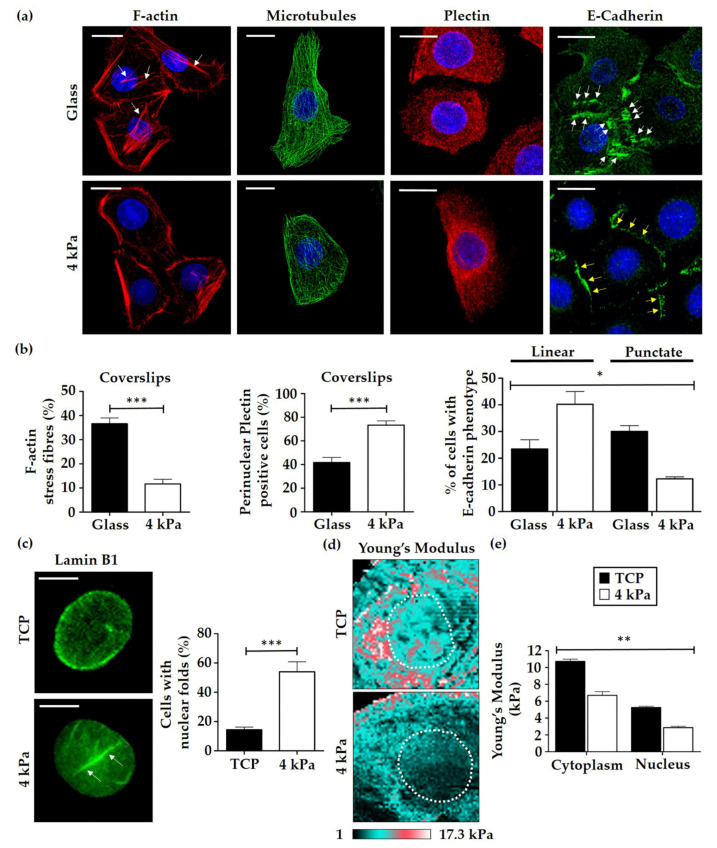
HEK cultured on BMH exhibited altered localisation for F-actin, plectin and E-cadherin and were observed to have softer cellular compartments. (**a**) Representative images of F-actin (arrows indicate stress fibres), microtubule, E-cadherin (white and yellow arrows indicate punctate and linear E-cadherin staining, respectively) and plectin arrangement in HEK cultured on glass and 4 kPa BMH coverslips. Scale bars = 20 µm. (**b**) Quantification of observed phenotypic differences in cytoskeletal and cytoskeletal-associated proteins in HEK on glass (Black columns) and 4 kPa BMH (White columns) coverslips. Data represent mean ±SEM, *n* = 3, statistical significance was assessed using an unpaired *t*-test (actin and plectin) and one-way ANOVA with Tukey’s post hoc test (E-cadherin), * *p* ≤ 0.05). (**c**) Representative images and quantification of visible nuclear abnormalities following exposure to osmotic shock in HEK cells primed on TCP and 4 kPa BMH cell culture dishes. Anomalies were characterised as visible folds in the nuclear envelope (see arrows). Scale bars: 10 µm. Data represent mean ±SEM, *n* = 3, statistical significance was assessed using an unpaired *t*-test, *** *p* ≤ 0.0001. (**d**) AFM force maps showing Young’s Modulus values of HEK primed on TCP and 4 kPa BMH. White dotted line highlights approximate location of nucleus based on correlating height map. (**e**) Quantification of average cytoplasmic and nuclear stiffness in HEK primed on TCP and 4 kPa BMH. Data represent mean ± SEM, statistical significance was assessed using one-way ANOVA with Tukey’s post hoc test, ** *p* ≤ 0.005.

To examine the direct impact of BMH on cell biology and biomechanics, the softest BMH substrate (4 kPa) was chosen for further analysis. HEK were cultured on glass and 4 kPa BMH coverslips for 4 days then immunostained for F-actin, microtubules, E-cadherin and the cytolinker plectin, all key cytoskeletal or cytoskeletal-associated proteins important in epidermal biology (Figure 3a). F-actin was observed to exhibit a decreased number of stress fibres in HEK cells on the softer 4 kPa substrates, while microtubules appeared unchanged between the two coverslips. The cytoskeletal-crosslinking protein plectin appeared as filamentous structures throughout the cytoplasm in HEK cultured on both TCP and 4 kPa BMH, but cells on the softer substrate had a notably common perinuclear localisation (Figure 3a). Quantification revealed that 73.3% of cells on 4 kPa BMH coverslips presented with perinuclear plectin, compared to 41.7% in HEK cultured on TCP (Figure 3b). E-cadherin, a vital component in ensuring the integrity of the epidermal barrier, was observed to be more punctate (Figure 3a, panel 4, white arrows) in HEK cultured on TCP, with the homophilic interaction of E-cadherin proteins in adjacent cells clearly distinguished. In contrast, HEK cultured on 4 kPa coverslips presented with an apparently more stable intercellular connection; the gaps between cells being less apparent and linear E-cadherin staining observed along the length of the membrane where adjoining cells were in full contact (Figure 3a, yellow arrows). Quantification of this difference in localisation revealed that when cultured on 4 kPa BMH, significantly more HEK exhibited junctional E-cadherin staining (Figure 3b), suggesting a potential for mature and tighter cell-cell connections, which is desirable in epidermal formation in vivo.

To investigate the direct impact of a softer cell culture substrate on HEK biomechanics at the subcellular level, the effects of nuclear stiffness were examined by performing a hyperosmotic shock assay. HEK cultured on glass and 4 kPa were exposed to a high concentration of sucrose (640 mM), then fixed and stained for the nuclear lamina protein lamin B1. The number of nuclear abnormalities, defined as clear folds in the nuclear envelope were quantified, and it was observed that 54.0% of HEK cultured on 4 kPa exhibited visible nuclear abnormalities compared to only 14.4% of HEK grown on glass (Figure 3c). This, therefore, suggests that HEK cultured on 4 kPa BMH had softer nuclei as they were more readily able to deform in response to osmotic shock. Consequently, atomic force microscopy (AFM) analysis was performed to provide quantitative Young’s Modulus values for HEK cultured on TCP and BMH substrates. Cells were primed on their respective substrates for 4 days then transferred to new TCP dishes to avoid the underlying substrate impacting the atomic force measurements. It was observed that HEK cultured on TCP were significantly stiffer at the cytoplasmic region and the region containing the nucleus than cells primed on 4 kPa BMH (Figure 3d,e), indicating that HEK are able to adapt their cellular stiffness in response to external rigidity and the associated mechanical cues.

### 3.3. The Expression of Relevant Mechanobiology Proteins Changes when HEK Are Cultured on Physiologically Relevant Substrates

**Figure 4:**
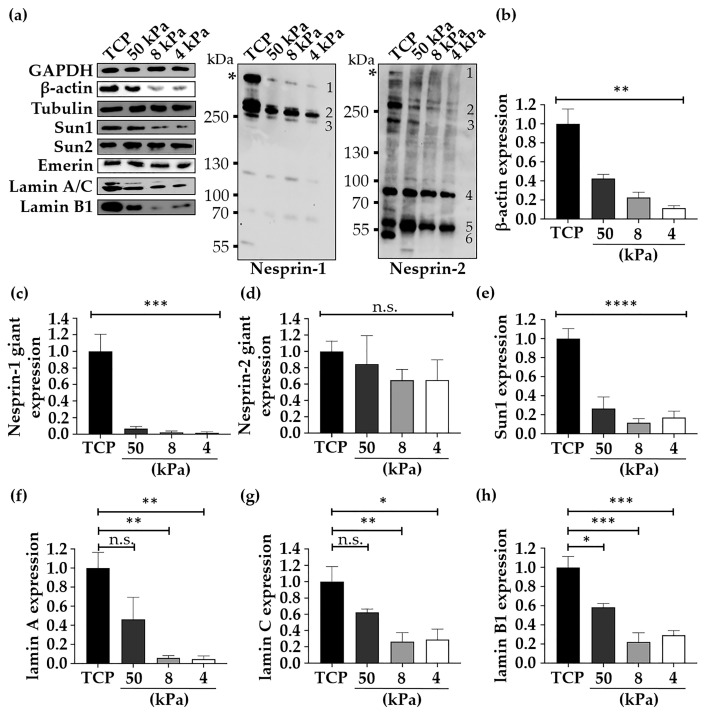
Expression levels of key mechanosensitive proteins were observed to change in HEK cultured on BMH. (**a**) Western blot analysis of LINC complex, cytoskeletal and nuclear lamina proteins in HEK cultured on TCP and BMH. GAPDH was used as the loading control. Asterisks denote the positions of the nesprin-1 and nesprin-2 giant isoforms (ABD-containing molecules; Band 1). The various isoforms detected by the nesprin-1 and nesprin-2 antibodies are numbered. (**b**–**h**) Quantification of the expression changes (relative expression levels normalised to TCP) in key LINC complex core and associated proteins in HEK cells cultured on BMH compared to the control TCP. Data represent mean ±SEM, *n* = 3. Statistical significance was assessed using one-way ANOVA with Dunnett’s post hoc test. Asterisks indicate statistical significance (* *p* ≤ 0.05, ** *p* ≤ 0.005, *** *p* ≤0.0005, **** *p* ≤ 0.00005); n.s. = non-significant.

Changes in the expression of proteins with key roles in mechanobiology were then documented. Specifically, western blotting was used to assess changes in the levels of key cytoskeletal, cytoskeletal-associated and LINC complex proteins (Figure 4a), with GAPDH used as the loading control. Tubulin (Figure S2), Sun2 (Figure S3), and emerin (Figure S4) proteins did not show a noticeable change in their levels in response to BMH culture. Most importantly, the proteins that were significantly downregulated across all BMH substrate ranges were β-actin (Figure 4b), nesprin-1 actin-binding domain (ABD)-containing isoforms (including nesprin-1 giant; 1 MDa) (Figure 4c), Sun1 (Figure 4e), lamins A/C (Figure 4f,g) and lamin B1 (Figure 4h). In contrast to nesprin-1 isoforms, which followed a similar pattern on BMH (Figure 4c and Figure S5), the expression profile for nesprin-2 proteins was more complex and heterogeneous. As the specific nesprin-1 and nesprin-2 isoforms that are expressed in epidermal cells have not been annotated yet, the protein bands detected using anti-nesprin western blotting will be referred to according to their migration (high molecular weight isoforms were labeled first; Figure 4a) and estimated molecular weights after SDS-PAGE. Slight downregulation trends were exhibited for the F-actin associated nesprin-2 giant isoform (800 kDa, band 1; Figure 4d), nesprin-2 ~260 kDa (Band 2; Figure S6), ~212 kDa (Band 3; Figure S6), and ~48 kDa (Band 6; Figure S6) isoforms in relation to the degree of substrate softness (Figure 4a). However, a trend towards upregulation was detected for nesprin-2 ~57 kDa (Band 5) isoforms, which was significant for cells grown on 50 kPa biomimetic dishes (Figure 4a and Figure S6). Altogether these results demonstrate that extracellular mechanics affect the expression of nesprin-1, nesprin-2, Sun1 and nuclear lamina proteins in HEK cells, which collectively highlights drastic changes in nuclear envelope proteome composition upon BMH cell culture. Moreover, the data suggest different roles for nesprin-1 and nesprin-2 proteins in keratinocyte mechanobiology based on their differential protein expression responses.

### 3.4. HEK Primed on 50 kPa BMH form Thicker and Well Organised Epidermal Models

In order to develop 3D epidermal tissue models in vitro, HEK were cultured on polycarbonate porous membranes to induce 3D epidermal assembly. One of the challenges of producing 3D skin models is that cells must first be cultured in a particularly unnatural 2D environment. To bypass this issue, HEK were primed on 4 kPa and 50 kPa BMH for 4 days prior to setting up epidermal models, and compared to models formed from TCP primed cells. Originally, models were generated using only TCP and 4 kPa primed cells, as 4 kPa demonstrated the clearest changes to cell architecture and behaviour in the previous experiments (Figure 1, Figure 2, Figure 3 and Figure 4). However, poor epidermal assembly in the 4 kPa primed models suggested that the substrate was too soft to promote the high level of proliferation that needs to take place during the submerged stage of 3D culture. As 8 kPa is still relatively soft, this stiffness was omitted from the 3D experiments and 50 kPa BMH were introduced.

**Figure 5:**
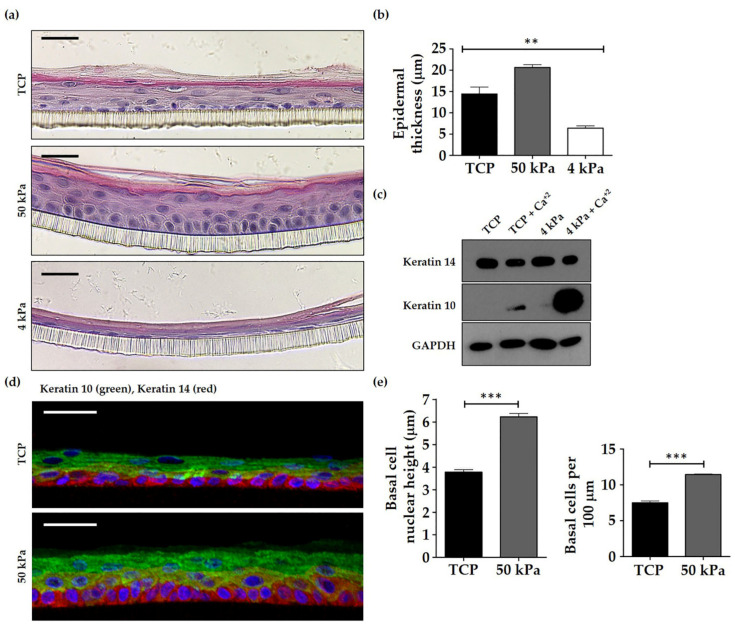
Assessment of epidermal equivalents formed from HEK primed on TCP and BMH. (**a**) Representative H and E images of epidermal equivalents cultured with HEK primed on TCP, 50 kPa and 4 kPa dishes respectively. Scale bars = 20 µm. (**b**) Quantification of observed epidermal thickness of models formed from HEK primed on TCP and BMH. Measurements were taken using ImageJ from the nucleated epidermal layers. Stratum corneum layers, characterised by the absence of nuclei were excluded. Data represent mean ±SEM, *n* = 3, statistical significance was assessed using one-way ANOVA with Tukey’s post hoc test, ** *p* ≤ 0.001. (**c**) Western blot analysis of keratin expression in high and low calcium conditions in HEK primed on TCP and 4 kPa BMH 2D cell culture dishes. GAPDH was used as the loading control. (**d**) Representative immunofluorescence images of epidermal equivalents expressing the epidermal markers keratin 14 (red) and keratin 10 (green). HEK were primed on TCP or 50 kPa dishes prior to model assembly. Scale bars = 20 µm. (**e**) Quantification of basal cell nuclear height (left graph) and cell number per 100 µm (right graph) in epidermal models constructed from TCP and 50 kPa BMH primed HEK. Data represent mean ±SEM, *n* = 3, statistical significance was assessed using an unpaired *t*-test, *** *p* ≤ 0.0001.

Histological analysis (Figure 5a) revealed that HEK primed on 50 kPa BMH formed models that appeared better organised and more similar to the in vivo epidermal appearance. There were well-defined basal and suprabasal layers, which were not observed as clearly in models made from TCP primed HEK. In contrast, HEK primed on 4 kPa did not assemble into good epidermal models, with only a single cell layer (nucleated cells) and thin stratum corneal layer visible. Quantification of the models (Figure 5b) revealed that 50 kPa HEK produced a significantly thicker epidermal model than either TCP or 4 kPa primed cells, suggesting that 50 kPa may better reflect the stiffness of the basement membrane that basal layer keratinocytes are exposed to in vivo. Interestingly, western blotting revealed a significant reduction of p63 levels in the 50 kPa BMH setting when compared to TCP, which suggests that HEK cells did not gain stem cell attributes (Figure S7). In contrast to 50 kPa, 4 kPa primed models were strikingly thinner than those generated with TCP primed cells. Western blot analysis of TCP vs. 4 kPa primed HEK (Figure 5c) showed that cells primed on 4 kPa BMH expressed a greater level of the epidermal differentiation marker keratin 10, particularly following supplementation with 1.5 mM CaCl_2_, as is used at the air-to-liquid interface (ALI) stage of culturing 3D models. Consequently, this suggests that very soft surfaces facilitate the premature differentiation of HEK cells, which impacts proper epidermal assembly.

Immunofluorescence analysis of models formed from TCP and 50 kPa HEK (Figure 5d) revealed that both keratin 14, a basal keratinocyte marker, and keratin 10 are expressed in the correct locations. However, the keratin 14 staining highlighted an apparent absence of cuboidal/columnar cells in the basal layer of models formed from TCP primed HEK, with the cells instead appearing flatter and atypical of the in vivo epidermis. In contrast, models formed from 50 kPa primed cells had a clearly defined basal layer and cells appeared either cuboidal and/or columnar with rounder overall nuclei. Quantification of the immunofluorescence data (Figure 5e) revealed that the height of the nuclei in the basal layer of the 50 kPa primed HEK models was significantly increased compared to the nuclei of TCP primed HEK cells. Furthermore, the number of basal cells per 100 µm was lower for TCP primed models than 50 kPa, suggesting that 50 kPa primed cells were less spread out, exhibiting the typical basal cell cuboidal/columnar phenotype seen in skin.

## 4. Discussion

The current study highlights the mechanosensitivity and mechanoresponsiveness of keratinocytes by demonstrating that distinct yet physiologically relevant extracellular mechanical cues differentially impact cell structure, the biomechanical properties of the nucleus and YAP1 localisation. The data presented is supported by and extends previous studies, which demonstrated that BMH cell culture induces significant changes in proliferation, migration, adhesion and cytoskeletal organisation [42,43,44].

Throughout this study, the mechanics of the native epidermal environment were imitated by culturing primary HEK on 4, 8 and 50 kPa BMH in comparison to conventionally used TCP, thus demonstrating how a stiff surface affects both cell biology and physiology. HEK were observed to have a greater cell density on softer substrates, particularly under high calcium conditions, and a uniform cobblestone morphology (Figure 1a,b). It has been previously noted that primary human keratinocytes cultured on TCP and even collagen I coated dishes had a heterogeneous morphology, whereas keratinocytes cultured on a fibroblast-derived matrix, mimicking the dermal ECM, had a cobblestone morphology [45], as witnessed in this present study. The increased cell density of BMH cell cultures was not attributable to a rise in levels of E-cadherin (Figure 1c and Figure S1) as first hypothesised due to its role as a core cell-cell adhesion protein in the epidermis [46]. Further investigation revealed that HEK on BMH exhibited decreased proliferation compared to those on TCP (Figure 1d,e), which led to the new hypothesis that the softness of BMH may be inducing terminal differentiation. This is in agreement with previous studies that observed keratinocyte terminal differentiation even under low calcium conditions with keratinocytes cultured on softer substrates [47,48]. Western blot analysis of the differentiation marker keratin 10 revealed that 4 kPa BMH induced an increase in expression, particularly under high calcium conditions (Figure 5c). Moreover, nuclear and cytoplasmic YAP1 levels were reduced in HEK grown on 4 kPa BMH (Figure 2b,c). The observed reduction of YAP1 staining on BMH confirms that the HEK were detecting the softer culture substrate and altering their mechanosensitive pathways accordingly. One striking observation was that relatively small changes in substrate stiffness, such as that between 4 kPa and 50 kPa, resulted in measurable differences in cell density (Figure 1b) and proliferation (Figure 1e). Together these data highlight the important impact that substrate stiffness can have on HEK cells in vitro, and suggest that culturing HEK on BMH promotes an in vivo-like cell and colony morphology, whilst prompting cells to exit the highly proliferative state induced by TCP.

Alterations in the organisation of cytoskeletal components were also observed in HEK grown on BMH. TCP primed cells exhibited a significantly greater number of stress fibres compared to BMH, with HEK on 4 kPa substrates displaying a cortical F-actin phenotype (Figure 3a,b). Actin reorganisation is a hallmark of keratinocyte terminal differentiation [49]; proliferative cells contain radially located short actin bundles, and terminally differentiated keratinocytes present with a well-developed circumferential actin network [50]. Another striking protein localisation change was observed in E-cadherin (Figure 3a), with HEK on TCP showing what appeared to be the start of trans-cadherin interactions between neighbouring cells, with a large amount of diffuse cytoplasmic staining still observed. In contrast, HEK on 4 kPa BMH appeared to be more closely bound to their neighbours, with E-cadherin staining more localised to the periphery of the cells. Thus HEK on softer substrates undergo epidermal sheet formation quicker than cells on TCP, which could promote epidermal assembly in a 3D setting. Given that the E-cadherin-α/β-catenin complexes of adherens junctions are bound to actin filaments [51], the increase in cortical F-actin structures observed on 4 kPa BMH could further explain the concentration of E-cadherin at the cell periphery.

In addition to the actin cytoskeleton, intermediate filaments (e.g., keratins), play a central role in epithelial cell mechanotransduction [52,53]. Rigid substrates increase keratin interconnections, the levels of disulfide-bonded multimers and keratinocyte stiffness [54]. Moreover, mutations in keratins decrease keratinocyte stiffness and adhesion by downregulating RhoA activity [55]. Keratins are bound to the nucleus via plectin, a cytolinker that has been shown to regulate keratinocyte nuclear morphology [56]. This present study indicates that HEK grown on 4 kPa BMH had a greater amount of perinuclear plectin staining than those on TCP. This coincides with current evidence suggesting that plectin protects against nuclear deformation in keratinocytes by limiting nuclear movement through the binding of keratin 14, which is subsequently reorganised to form a perinuclear network [57,58]. Plectin knockouts have been shown to have weakened perinuclear keratin 14 structures due to slight inhibition of the assembly of high molecular weight keratin 14 species, thereby putting the nucleus at greater risk of deformation as a result of mechanical forces subjected to the cell [56]. The osmotic shock assay and AFM analysis suggest that the nuclei of HEK on 4 kPa BMH are softer than those on TCP. Whilst no studies appear to have investigated the direct relationship between human keratinocyte colony density and mechanical pressure on nuclei, there is evidence that during epithelial expansion, polarisation gradients within a monolayer induce internal mechanical stress [59], and disruption of cell-cell junctions has highlighted the importance of intercellular adhesion in the generation of monolayer stress transmission [60]. Given the apparent increase in mature cell-cell junctions in response to culture on 4 kPa BMH (Figure 3a,b), the perinuclear plectin localisation we observed may be attributed to an increased need for a stable keratin network to protect the nucleus from mechanical stress transmitted via inter-/trans-cellular adhesion molecules.

Following the observed softening of HEK cells upon culture on 4 kPa BMH the expression levels using western blotting of key proteins were investigated. Whilst changes to tubulin expression were inconclusive, it was observed that β-actin levels decreased in response to culture on BMH, alongside key core LINC and LINC-associated components such as lamins A/C and B1. This present data indicate that in addition to A-type lamins, lamin B1 expression is drastically affected by keratinocyte mechanobiology modulation. A-type lamin level reduction would imply a softening of the nucleus, given the prominent role in providing its structural framework [61]. It is well known that remodelling of the nucleus, particularly expression of lamin A/C plays a role in regulating epidermal differentiation [62,63,64], with lamin-null mice exhibiting a thickened epidermal layer that is attributed to precocious differentiation [21]. In skin, lamin A is found in the epidermal suprabasal layers, while lamin C is expressed in both basal and suprabasal layers [64]. In contrast, lamin B1 is expressed in all epidermal layers [65]. Therefore, the downregulation of these proteins in cells grown on softer substrates cannot be explained by the shift towards differentiation hinted at by the changes in actin organisation (Figure 3a,b) and increased keratin 10 expression (Figure 5c) observed in HEK cultured on 4 kPa BMH.

With respect to LINC complex core components, it appeared that BMH cell culture affects both nesprins-1/-2 as well as their luminal binding partners, i.e., the Sun-domain proteins. Surprisingly, reductions were noted in Sun1 but not Sun2 protein levels. As Sun2 and emerin expression was largely unaffected despite a drastic change in nesprins-1/-2 isoform levels, it is suggested that the molecular changes are more pronounced at the outer nuclear membrane rather than the inner nuclear membrane following short BMH cell culture (e.g., 4 days). The concept that cell morphology and physiology changes are mirrored by the cytoskeleton-associated nesprins rather than the Suns, is not new. During muscle differentiation, and specifically during the transition from the single-nucleated myoblast to the multinucleated myotube, it is the nesprins that switch isoforms rather the Sun-domain proteins [66]. In the present study, nesprins-1 and -2 exhibited variable changes in protein level depending on the isoform that was examined. Yet, it was apparent from the generated data that in particular, nesprin-1 ABD-containing isoforms including the 1 MDa nesprin-1 were reduced in BMH conditions. Whether the downregulation of these nesprins is solely responsible for the actin cytoskeleton reorganisation on softer substrates requires further research. Nesprins define the peri-nuclear cytoskeletal landscapes in keratinocytes [67]. However, the upregulation of specific low molecular weight nesprin-2 proteins (i.e., 57 kDa) upon BMH usage suggests a more complex molecular mechanism. To provide detailed mechanistic insights and to be able to link specific nesprin molecules to certain cytoskeletal re-arrangements upon extracellular biomechanical modulation, a systematic nesprin proteome analysis is essential. In particular, understanding how mechanical forces affect the expression of nesprins and their associated proteins including Sun1 will be key. Do cytoskeleton-mediated mechanical forces modulate nesprin tertiary structure and post-translational modifications that destabilise or stabilise specific nesprin isoforms? Additionally, which are the molecular adaptations that occur first and how do they impact LINC complex composition/structure and the nuclear lamina?

In addition to alterations in LINC components, a decrease in actin expression would correlate with the lower cytoplasmic rigidity observed in the current AFM investigations. The cytoskeleton is a predominant factor in controlling cell stiffness [68], and cells have been shown to adapt their stiffness to match soft elastic substrates by altering actin crosslinking [69]. Consequently, rather than being a result of differentiation, the changes observed in LINC expression could be a direct result of the reduced mechanical stimuli provided by BMH and the subsequent adaption of the cell to mimic substrate stiffness. This would create a feedback loop, whereby reduced external tension on the cytoskeleton resulted in reduced pull on the nucleus, leading to downregulation of cytoskeletal and LINC components to “soften” the cell, which would then further reduce nuclear tension. It has been well established in recent years that the LINC complex plays a key role in regulating the transcriptomic response to mechanical stimuli [70,71,72]. Therefore, it is plausible that changes in LINC complex expression drive epidermal stratification and differentiation, rather than the LINC components altering in response to the differentiated phenotype observed in HEK cells on 4 kPa substrates. The aforementioned concept is further supported by the recent patent application (United States Patent Application 20190352605), where LINC complex disruption and the consequential softening of the nuclei/cells facilitates the stratification of keratinocytes, and the engineering of high-quality epidermal models is demonstrated. Similarly, β1 integrin-dependent tension is exerted on the LINC complex, which represses epidermal differentiation in mouse keratinocytes. As a consequence, loss of both Sun1 and Sun2 in mice increases epidermal thickness [9].

Culturing HEK on TCP, 50 kPa BMH and 4 kPa BMH revealed that cells primed on 50 kPa BMH provided the best conditions for optimal epidermis formation (Figure 5a). H and E staining showed that HEK cultured on TCP prior to model set up produced a relatively disorganised epidermis with an indistinct basal layer. In contrast, HEK primed on 50 kPa BMH produced models that were thicker and better organised, demonstrating a clear layer of basal cells and appropriate stratification. Cells primed on 4 kPa BMH produced the most compromised epidermis, with only one layer of flattened cells that did not resemble the cuboidal morphology expected of an epidermal basal layer.

Altogether, the current study demonstrates that extracellular mechanics play major roles in HEK cell biology, architecture and physiology, which can be potentially exploited in tissue engineering. Our data favour the mechanical memory concept [25,26] as biomechanically primed HEK cells retained their cellular attributes despite the prolonged 3D cell culture (16 days). Engineering skin models in vitro is a costly and time-consuming process. Future strategies may capitalise on these current findings and engineer epidermal tissue by either pre-conditioning HEK cells on different 2D BMH stiffness ranges first (e.g., 4 and 50 kPa) or by using stratified 3D scaffolds, which display the desired stiffness values. For the pre-conditioning approach, biomechanically primed HEK cells could be sequentially combined in a 3D cell culture setting, e.g., formation of basal layers using 50 kPa pre-conditioned cells, which are then topped-up with 4 kPa primed HEK cells, in order to build efficiently high-quality skin models. Whether the keratinocyte mechanobiology insights can be translated to in vivo situations such as in skin wounding is worth exploring.

## 5. Conclusions

This study advances the current understanding of the molecular and structural changes that occur within cells when grown on substrates mimicking the stiffness of native skin. It has been demonstrated that keratinocytes grown on TCP exhibit increased cell proliferation and cellular stiffness. In terms of expanding keratinocyte cell numbers quickly in vitro, TCP substrates are advantageous. However, the cultured cells do not perform as well compared to cells that were previously primed on 50 kPa stiff substrates when it comes to engineering an epidermal model in vitro. Surprisingly, even short-term cell culture (i.e., 4 days) on BMH impacts cellular architecture, LINC complex molecular composition, and the mechanical properties of the nucleus. The growth of cells on soft 4 kPa substrates yields softer cells, which might harbor softer nuclei. The expression of the nesprin-1/-2 high molecular weight isoforms (>200 kDa), Sun1 and nuclear lamina constituents are downregulated when cells are grown on soft biomimetic substrates, which together may explain a reduction in nuclear stiffness.

In relation to the nesprins, it is suggested that the observed changes in their expression are key in determining the cytoskeletal landscapes of cells grown on BMH. Finally, the enhanced mechanoresponsive sensitivity of keratinocytes is highlighted, as cells elicited differential cellular responses when grown on 4, 8 and 50 kPa stiff substrates. This is surprising for such a specialised, keratin-rich and resilient epithelium, which is known for endowing mainly protective functions upon the upper layers of skin. As evidenced in the current study, extracellular biomechanics affects both the cell biology and physiology of keratinocyte cells. Therefore, it is predicted that harnessing cell biomechanics will be beneficial for tissue engineering, treatment of skin disease and wound healing.

## Data Availability

The data presented in this study are available on request from the corresponding author.

## Supplementary Materials

The following are available online at https://www.mdpi.com/article/10.3390/cells10051177/s1, Figure S1: E-cadherin expression levels in HEK cells grown on TCP and 4 kPa coverslips. Figures S2-S6 supplement the western blot data of Figure 4a. Figure S2: Relative β-tubulin protein expression levels in cells grown on TCP and BMH cell culture dishes, Figure S3: Relative Sun2 protein expression levels in cells grown on TCP and BMH cell culture dishes, Figure S4: Relative emerin protein expression levels in cells grown on TCP and BMH cell culture dishes, Figure S5: Relative nesprin-1 isoform expression levels in cells grown on TCP and BMH cell culture dishes, Figure S6: Relative nesprin-2 isoform expression levels in cells grown on TCP and BMH cell culture dishes. Figure S7: Relative p63 expression levels in cells grown on TCP and 50 kPa BMH cell culture dishes. Table S1: List of primary antibodies, Table S2: List of secondary antibodies.
