# Supplementary material for: Culturing Keratinocytes on Biomimetic Substrates Facilitates Improved Epidermal Assembly In Vitro
Source: Cells. 2021 May 12;10(5):1177. doi: 10.3390/cells10051177 (PMC8151809; doi:10.3390/cells10051177)
Supplement: Supplementary file 1 [file cells-10-01177-s001.zip › cells-1182646-supplementary.pdf]

Supplementary Materials:

Table S1. Primary antibodies used for the immunostaining of cells cultured in 2D or 3D environments.

Antibody	Supplier	Product Code	Species	Dilution
**YAP1**	Novus Biologicals	NB-110-58358	Rabbit	1:50
**Tubulin**	WA3, kind gift of Dr. U. Euteneuer	-	Mouse	1:50
**E-cadherin**	Abcam	ab1416	Mouse	1:100
**Plectin (C-20)**	Santa Cruz Biotechnology	sc-7572	Goat	1:200
**Lamin B1**	Abcam	ab16048	Rabbit	1:400
**Cytokeratin 10**	Abcam	ab76318	Rabbit	1:100
**Cytokeratin 14**	Abcam	Ab7800	Mouse	1:100

Primary antibodies used in western blotting.

Antibody	Supplier	Product Code	Species	Dilution
**GAPDH**	Calbiochem	CB1001	Mouse	1:5,000
**Cytokeratin 10**	Abcam	ab76318	Rabbit	1:5,000
**Cytokeratin 14**	Abcam	Ab7800	Mouse	1:5,000
**β-actin**	Sigma-Aldrich	AC-74	Mouse	1:5,000
**Tubulin**	WA3, kind gift of Dr. U. Euteneuer	-	Mouse	1:5,000
**Emerin**	Vector Laboratories	VP-E602	Mouse	1:500
**Lamin A/C**	ImmuQuest	IQ332	Mouse	1:50
**Lamin B1**	Abcam	ab16048	Rabbit	1:500
**Sun1**	Abcam	ab124770	Rabbit	1:500
**Sun2**	Abcam	EPR6557	Rabbit	1:500
**E-cadherin**	Abcam	ab1416	Mouse	1:1000
**p63**	Abcam	ab124762	Rabbit	1:1,000
**Nesprin-1**	In-house[1]	N-ABD	Rabbit	1:500
**Nesprin-2**	In-house[2]	PABK1	Mouse	1:1,000

References:

1. Padmakumar, VC.; Abraham, S.; Braune, S.; Noegel, A.A.; Tunggal, B.; Karakesisoglou, I.; Korenbaum, E. Enaptin, a giant actin-binding protein, is an element of the nuclear membrane and the actin cytoskeleton. *Exp Cell Res* **2004**, *295*, 330-339. doi: 10.1016/j.yexcr.2004.01.014.

2. Libotte, T.; Zaim, H.; Abraham, S.; Padmakumar, VC.; Schneider, M.; Lu, W.; Munck, M.; Hutchison, C.; Wehnert, M.; Fahrenkrog, B.; Sauder, U.; Aebi, U.; Noegel, A.A.; Karakesisoglou, I. Lamin A/C dependent localization of Nesprin-2, a giant scaffolder at the nuclear envelope. *Mol Biol Cell* **2005**, *16*, 3411–3424, doi: 10.1091/mbc.e04-11-1009.

Table 2. Secondary antibodies and other fluorescent agents used for the immunostaining of cells cultured in 2D or 3D environments.

Antibody	Supplier	Product Code	Species	Dilution
**Phalloidin (Alexa Fluor™ 568)**	Invitrogen	A12380	-	1:1,000
**Anti-Mouse (Alexa Fluor™ 488)**	Invitrogen	A21202	Donkey	1:1,000
**Anti-Mouse (Alexa Fluor™ 568)**	Invitrogen	A11004	Goat	1:1,000
**Anti-Rabbit (Alexa Fluor™ 488)**	Invitrogen	A21441	Chicken	1:1,000
**Anti-Rabbit (Alexa Fluor™ 568)**	Invitrogen	A11036	Goat	1:1,000
**Anti-Goat (Alexa Fluor™ 555)**	Invitrogen	A21432	Donkey	1:1,000
**Secondary antibodies used in western blotting:**				
**Anti-Mouse POD**	Sigma-Aldrich	A4416	Goat	1:10,000
**Anti-Rabbit POD**	Sigma-Aldrich	A6154	Goat	1:5,000

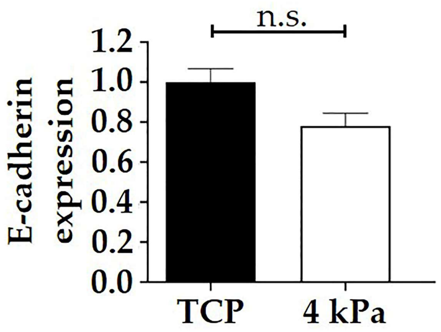

Figure S1. E-cadherin expression is unaffected in HEK cells grown on TCP and 4 kPa BMH substrates. Quantification of relative E-cadherin expression levels in HEK cells cultured on TCP and 4 kPa BMH cell culture dishes for 4 days. All indicated values were normalised to TCP (data represent mean ±SEM, $n = 3$). Statistical significance was assessed using an unpaired t-test, *** $p \leq 0.0001$.

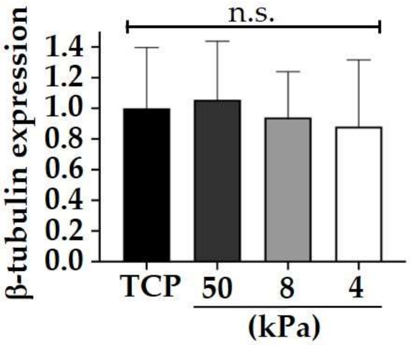

Figure S2. β-tubulin expression is unaffected in HEK cells grown on TCP and BMH substrates. Quantification of relative β-tubulin expression levels in HEK cells cultured on TCP and BMH cell culture dishes for 4 days. All indicated values were normalised to TCP (data represent mean ±SEM, $n = 3$). Statistical significance was assessed using one-way ANOVA followed by Dunnett's post hoc test, n.s.= non-significant.

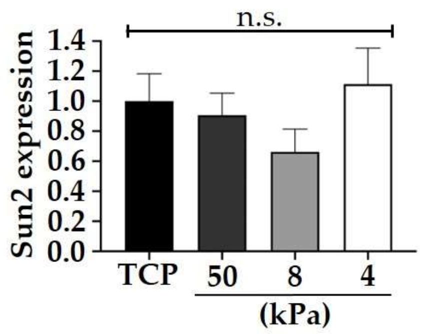

Figure S3. Sun2 expression is unaffected in HEK cells grown on TCP and BMH surfaces. Quantification of relative Sun2 expression levels in HEK cells cultured on TCP and BMH cell culture dishes for 4 days. All indicated values were normalised to TCP (data represent mean ±SEM, n = 3). Statistical significance was assessed using one-way ANOVA followed by Dunnett's post hoc test, n.s.= non-significant.

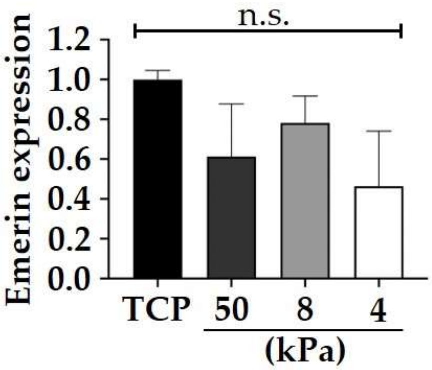

Figure S4. Emerin expression is unaffected in HEK cells grown on TCP and BMH surfaces. Quantification of relative emerin expression levels in HEK cells cultured on TCP and BMH cell culture dishes for 4 days. All indicated values were normalised to TCP (data represent mean ±SEM, n = 3). Statistical significance was assessed using one-way ANOVA followed by Dunnett's post hoc test, n.s.= non-significant.

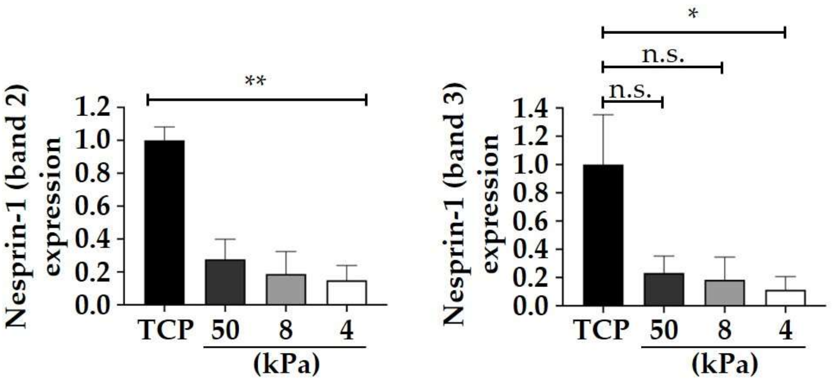

Figure S5. Specific nesprin-1 isoforms are downregulated in HEK cells grown on BMH cell culture dishes. Quantification of relative nesprin-1 isoform (band 2 [~267 kDa] and band 3 [~250 kDa]) expression levels in HEK cells cultured on TCP and BMH cell culture dishes for 4 days. All indicated values were normalised to TCP (data represent mean ±SEM, n = 3).

Statistical significance was assessed using one-way ANOVA followed by Dunnett's post hoc test. Asterisks indicate statistical significance (* = p≤0.05, ** = p≤0.005); n.s.= non-significant.

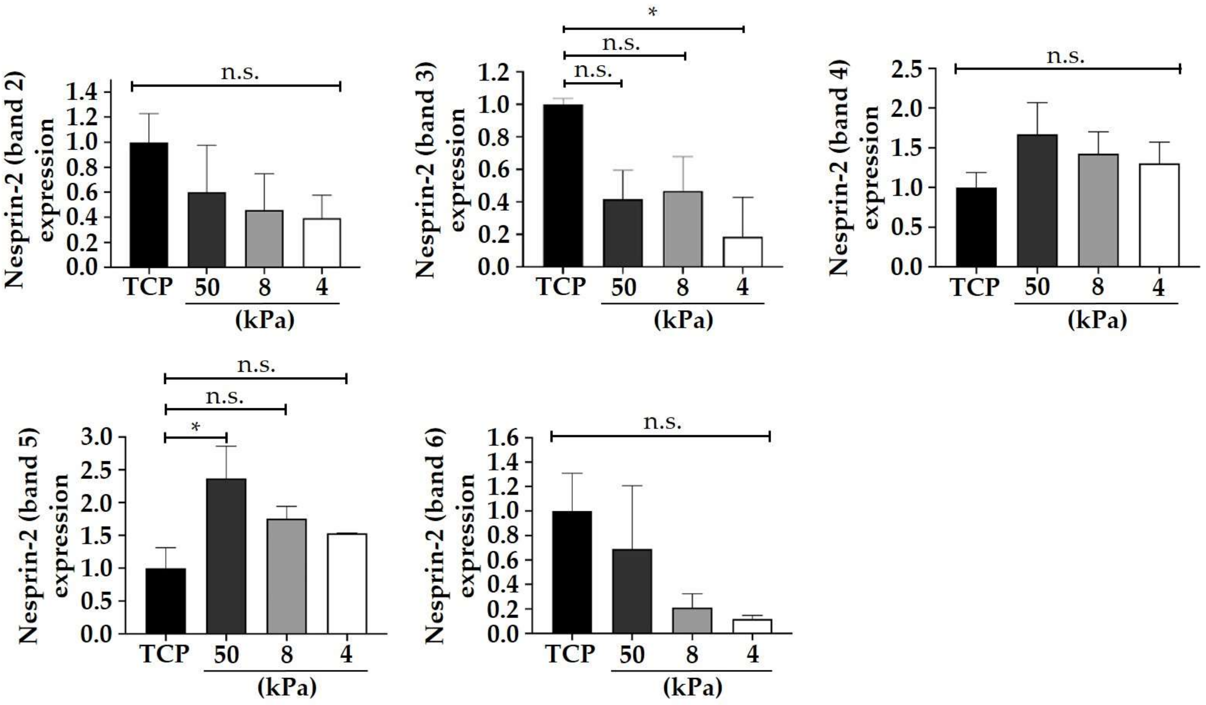

Figure S6. The effects on nesprin-2 expression upon BMH cell culture in HEK cells are complex and isoform specific. Quantification of relative nesprin-2 isoform (bands 2–6) expression levels in HEK cells cultured on TCP and BMH cell culture dishes for 4 days. All indicated values were normalised to TCP (data represent mean ±SEM, n = 3). Molecular weight estimations of nesprin-2 isoforms based on SDS-PAGE: Band 2, ~260 kDa; band 3, ~212 kDa; band 4, ~83 kDa; band 5, ~57 kDa; band 6, ~48 kDa. Statistical significance was assessed using one-way ANOVA followed by Dunnett's post hoc test. Asterisks indicate statistical significance (* $p \leq 0.05$); n.s.= non-significant.

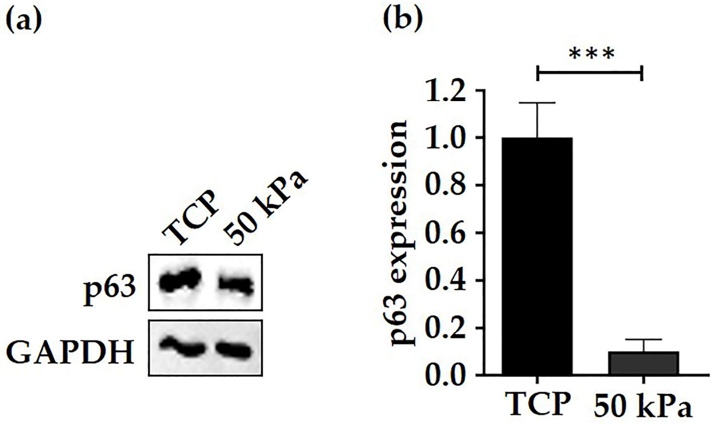

Figure S7. The expression of p63 is reduced in HEK cells grown on 50 kPa BMH substrates. (a) Western blot of p63 expression in HEK cultured on TCP and 50 kPa BMH cell culture dishes. GAPDH levels indicate equal loading of proteins. (b) Quantification of relative p63 expression levels in HEK cells cultured on TCP and 50 kPa BMH cell culture dishes for 4 days. All indicated values were normalised to TCP (data represent mean ±SEM, n = 3). Statistical significance was assessed using an unpaired t-test, *** p ≤ 0.0001.
